# Gratitude practice helps undergraduates who experienced an earthquake in China find meaning in life

**DOI:** 10.1038/s41598-024-61256-3

**Published:** 2024-05-13

**Authors:** Xueli Cai, Ningyi Zhou, Junpeng Chen, Zhuozhu Mao, Shujun Wang, Zaibing Luo, Mei Xie, Yanhui Mao

**Affiliations:** 1 Institute of Applied Psychology, Psychological Research and Counseling Center, Southwest Jiaotong University, Chengdu, China; 2 School of Social and Behavioral Sciences, Nanjing University, Nanjing, China; 3 Institute of Education, University College London, London, UK; 4 School of Foreign Languages, Southwest Jiaotong University, Chengdu, China; 5 Department of Social and Developmental Psychology, Sapienza University of Rome, Rome, Italy

**Keywords:** Gratitude, Learning engagement, Meaning in life, University students, Earthquake, Psychology, Human behaviour

## Abstract

This study was conducted following a magnitude 6.8 earthquake that occurred in early September 2022, coinciding with the commencement of a positive psychology course for the affected students. A sample of 479 Chinese undergraduates was recruited for an intervention focused on weekly gratitude practice. Data were collected through an online questionnaire package at 3 time points: the first week of the course (Time 1), the fifth week (Time 2), and the ninth week (Time 3), assessing gratitude, learning engagement, and the meaning of life. Findings revealed that gratitude significantly predicted meaning in life through learning engagement over time. This highlights the significant mediating role of learning engagement in the context of earthquakes and provides insights for positive interventions aimed at facilitating personal growth among emerging adults in higher educational settings, particularly those who have experienced traumatic events such as earthquakes.

## Introduction

On September 5, 2022, Sichuan Province in China experienced an earthquake of magnitude 6.8 (Sichuan Earthquake Administration: https://www.scdzj.gov.cn/xwzx/sczx/202209/t20220905_53309.html). Considering the substantial psychological impact of earthquakes on university students, particularly in terms of post-traumatic stress disorder symptoms and disruptions to their sense of meaning in life (MIL)^1^, it is crucial to explore effective interventions that can enhance MIL. The significance of cultivating MIL is highlighted by its association with improved psychological adjustment^2^ and enhanced well-being^3^, which are vital for assisting students in coping with the challenges arising from natural disasters like earthquakes. Therefore, as the students affected by the earthquake were welcoming a new academic year and the start of their elective positive psychology course, proper interventions were implemented, such as gratitude practices integrated within their existing positive psychology course.

Gratitude has been longitudinally studied as a mechanism to attenuate symptoms among Chinese adolescents following earthquakes^4–7^. In the realm of nurturing meaning in life (MIL), gratitude practice has emerged as a noteworthy element. Specifically, practices encouraging students to think gratefully (e.g., writing gratitude letters or listing things they feel grateful for^8–10^) are commonly adopted to intervene. Previous empirical investigations have shown that gratitude not only directly boosts college students’ MIL, but also indirectly affects them through mediating variables^11,12^.

While the direct effect of gratitude on MIL is well acknowledged, demonstrating its utility in aiding students affected by distress, the potential mediating role of learning engagement (LE) in this relationship has yet to be thoroughly investigated. This is particularly relevant during times of trauma as an individual’s involvement or quality of effort plays a central role in determining the extent and nature of development and learning at university. In traumatic contexts, students may face disrupted routines and heightened emotional challenges, making engagement a critical factor in maintaining continuity and normalcy in their academic and personal lives. Prior research underscores that gratitude positively influences academic engagement among Chinese university students, fostering resilience against the academic disengagement often triggered by stressful events^13,14^. More specifically, gratitude has been shown to prevent a decline in academic engagement among Chinese adolescents over time following an earthquake^15^. By actively engaging in their educational pursuits and remaining motivated to achieve desired outcomes, students may derive a greater sense of purpose and significance in their lives, even in adversity^16^. This enhanced focus on engagement during traumatic times helps buffer against the negative impacts of stress and trauma, supporting students’ overall well-being and academic success.

Therefore, studying how to improve MIL through gratitude practice and understanding the potential mediating mechanism of LE is of great theoretical and practical importance. However, to the best of our knowledge, empirical studies examining the interplay between gratitude, LE, and MIL among university students in stress-induced scenarios such as earthquakes remain limited. To this end, this study seeks to address this research gap and provide insights into the potential pathways through which gratitude and LE can strengthen university students’ MIL amid the challenges posed by the earthquake.

### Gratitude and meaning in life

Gratitude is defined as “*a sense of thankfulness and joy in response to receiving a gift, whether the gift is a tangible benefit from a specific other or a moment of peaceful bliss evoked by natural beauty*”^17^, p. ^554^. It reflects a positive outlook toward life, emphasizing the positive aspects of the world^18,19^. In contrast to negative orientations, gratitude represents a stable and positive psychological trait that may serve as a stable predictive factor for developing post-traumatic growth following earthquakes.

Meaning in life (MIL) is characterized as a profound sense that one’s life has purpose and meaning, along with a feeling of fitting into the grand scheme of things^20^. It varies based on individual values and life experiences^21^, encompassing a sense of purpose and significance derived from fulfilling one’s core needs for self-worth, efficacy, and value^3^.

Extensive research has established a connection between gratitude and MIL. For instance, gratitude has been associated with positive affect, concern for personal and others’ welfare, and better adjustment outcomes, ultimately contributing to a greater sense of MIL^17,18, 22^. In university students, gratitude has been shown to both directly and indirectly predict MIL^23^. Expressing gratitude, such as writing gratitude notes, has also enhanced MIL^24^.

The “find-remind-and-bind” theory proposed by Algoe^25^ offers a plausible explanation for how gratitude predicts an increase in MIL. This theory suggests that gratitude enables individuals to recognize and appreciate the positive actions of others (find), recall these actions and the individuals who performed them (remind), and cultivate closer relationships and connections with these individuals (bind). These strengthened relationships and connections can contribute to a heightened sense of belongingness and relatedness, enrich the negotiation of social relationships, foster adult responsibility, and ultimately, crystallize adult identity and life’s purpose, which are essential sources of MIL^26,27^.

Building upon the existing theoretical and empirical literature, this study hypothesizes that gratitude can predict an individual’s MIL after the experience of an earthquake. Furthermore, it is essential to investigate potential mediators that may help elucidate the underlying mechanisms through which gratitude influences MIL (e.g., the mediation of learning engagement).

### Learning engagement as a mediator

Learning engagement (LE) refers to the quality of the effort students devote to educationally purposeful activities that contribute directly to the desired outcomes^28^. With its vigor, dedication, and absorption dimensions, it plays a crucial role in a student’s academic journey and has been of interest to researchers studying positive psychology and well-being in the educational context^29–31^.

Previous implicit research suggests that gratitude could enhance MIL by promoting LE, and several theoretical perspectives have informed this connection. First, the Self-Determination Theory (SDT)^32,33^ offers a crucial framework for understanding this relationship. According to SDT, fulfilling basic psychological needs, such as autonomy, competence, and relatedness, fosters an individual’s active engagement in behaviors, including learning behaviors. Gratitude, which has been found to bolster the sense of relatedness to others, can create a more engaging and supportive learning environment, enhancing the perception of life as meaningful^25,34^.

In addition, the broaden-and-build theory^35,36^ suggests that positive emotions broaden people’s mindsets and build personal resources that encourage further reciprocal behaviors. Gratitude harnesses individual motivation, along with other pro-social behaviors triggered by gratitude practice^37^. This motivation, combined with increased vigor and dedication, can lead students to be more devoted to their studies, ultimately contributing to a strong sense of MIL^24^.

While these theories suggest that LE is widely recognized as a mediator in educational psychology, its specific role between gratitude and MIL in trauma recovery is an innovative contribution to the field. By actively engaging in their learning experiences and investing their resources in academic pursuits, students may find a deeper sense of purpose and significance in their lives, particularly in the aftermath of challenging events such as earthquakes.

### The present study

This study was initiated in the aftermath of a 6.8 magnitude earthquake at a local university in early September 2022. The disaster coincided with the commencement of a positive psychology course, offering a unique opportunity to explore the potential benefits of gratitude practice in a traumatized student population. To address the gap in research concerning the longitudinal impact of gratitude on MIL and the mediating role of LE, this study adopted a three-wave longitudinal design. Specifically, two hypotheses were proposed: H1—Gratitude is positively associated with MIL longitudinally; H2—LE mediates the relationship between gratitude and MIL.

## Methods

Taking advantage of the longitudinal design, which provides valuable information about temporal precedence and allows for the examination of cause-and-effect relationships^38^, we adopted a three-wave longitudinal mediation design to investigate the temporal causal relationship between gratitude and MIL, as well as the potential mediating mechanism of LE. Three study variables, Gratitude, LE, and MIL, were measured three times. Then a cross-lagged panel model (CLPM), regarded as the most popular model for analyzing the longitudinal data with mediation^39^, was established and evaluated to test the hypotheses.

### Participants

The city of Chengdu, where many of our participants reside, was significantly affected by the earthquake while still under lockdown due to pandemic restrictions. During this time, residents, including our participants, experienced notable disruptions. Distress was evident in social media posts from residents, with reports of shaking chandeliers and spilled fish tanks. Additionally, Sichuan was grappling with an unusual heatwave and subsequent power shortages, intensifying stress levels during this period. As the power shortage was beginning to ease, Chengdu faced a surge in COVID-19 infections. Within a week, the city reported nearly 800 confirmed cases. On September 1st, an official notice was issued in Chengdu requiring all residents to stay home from 6 PM onwards. This notice prompted a large-scale mobilization of residents, resulting in long queues forming at supermarkets. Street vendors quickly sold out large quantities of vegetables as they were purchased by the populace. These overlapping and cascading events underscore the multifaceted traumatic conditions experienced by our participants, reinforcing our exploration of gratitude as a buffer against stress.

**Figure 1:**
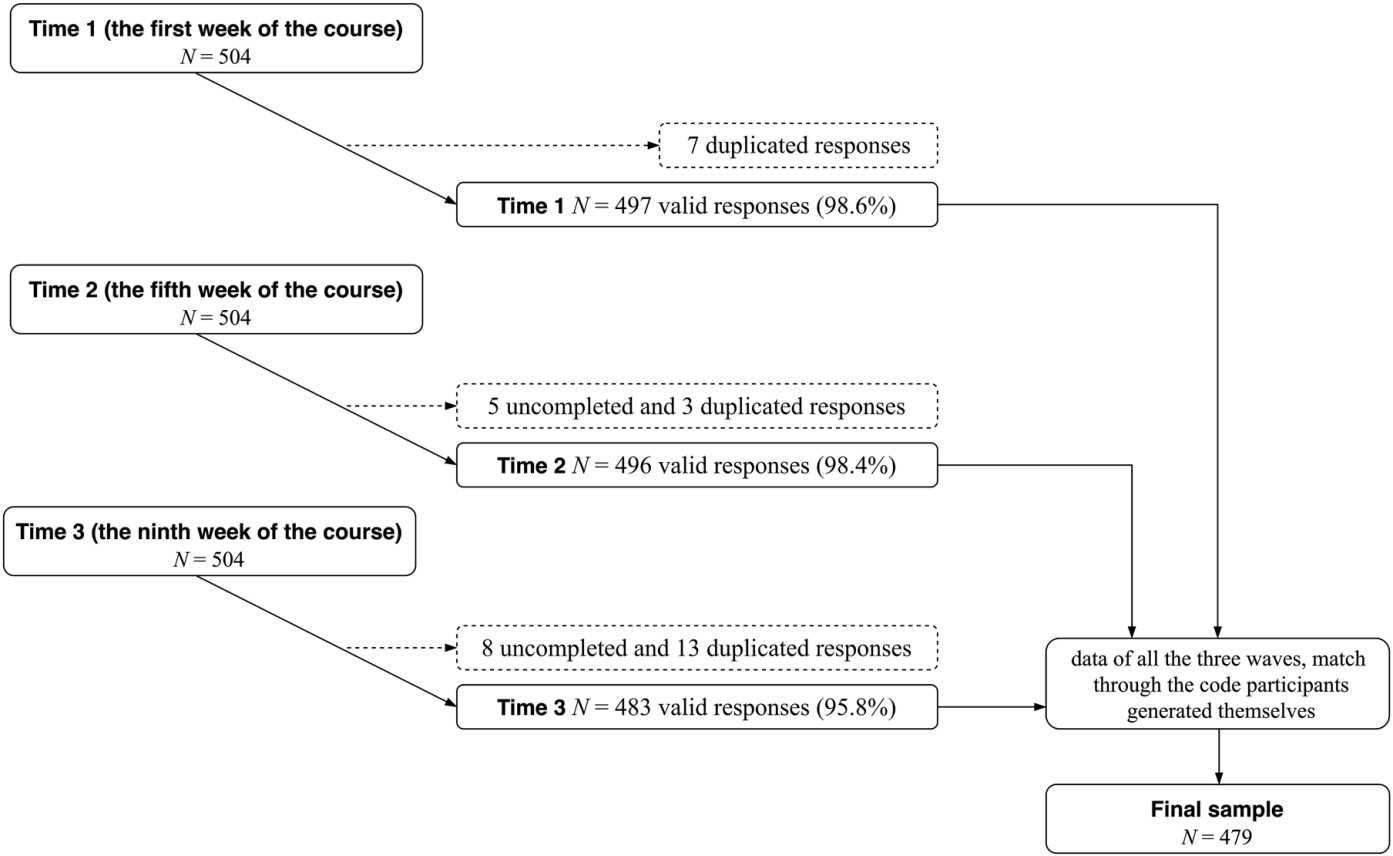
Data collection phases.

Utilizing the Convenience Sampling Method, 504 undergraduate students from a university in southwest China were initially recruited with informed consent detailing the study’s purpose, voluntary participation, and assurance of confidentiality. All participants took part in a positive psychology course conducted by the corresponding author. Data were collected at three time points. Ultimately, 479 participants (228 females and 251 males, representing a 95.0% overall retention rate) of full participation (completing the questionnaire package at all 3 time points) constituted the final sample (see Fig. 1) whose data will be taken into statistical analysis. The participants, from freshmen to seniors with a mean age of 19.84 (*SD* = 1.02, Range = 18–25), come from different majors (48.9% from Engineering, 30.1% from Literature, 17.1% from Science and Technology, and 3.9% from other majors), representing a diverse sample of varying backgrounds in majors.

### Materials

#### Measures

Three study variables, gratitude, LE, and MIL, were measured through three questionnaires, respectively. To ensure their validity as well as applicability, these questionnaires underwent a rigorous two-step translation and back-translation process^40^ by two professors with both English and psychology backgrounds.

##### Gratitude

Gratitude was measured using the Gratitude Questionnaire-Six Item Form (GQ-6)^41^ due to its comprehensive assessment of gratitude’s frequency, intensity, density, and span^18^, as well as its well-documented and robust reliability and construct validity (e.g., Refs.^41–43^). Participants were required to rate the six statements (e.g., “If I had to list everything that I felt grateful for, it would be a very long list”) on a Likert scale ranging from 1 (“strongly disagree”) to 7 (“strongly agree”). A higher rating score represented a higher level of gratitude. However, in the present study, akin to the findings in previous findings (e.g., Refs.^22,44^), item 3 (“When I look at the world, I don’t see much to be grateful for”) and item 6 (“Long amounts of time can go by before I feel grateful to something or someone”) yielded low factor loadings and were thus excluded from further analyses^45^. The internal consistency reliability estimated by Cronbach’s alpha coefficient for the final 4-item scale was 0.85, 0.94, and 0.95 at T1, T2, and T3, respectively.

##### Learning engagement

The 6-item version of the Utrecht Work Engagement Scale (UWES), revised by Ouweneel et al.^46^, was adopted to assess learning engagement. This version, derived from the original 9-item scale developed by Schaufeli et al.^47^, encompasses three subscales: vigor, dedication, and absorption. The UWES has demonstrated good psychometric properties in previous studies (e.g., Ref.^48^). In the present study, to better align the 6-item version with the educational context, any terms related to work or job were replaced with the term “study” (e.g., “I felt energetic in my study”, “I was inspired by my study”, and “I was completely absorbed in my study”). Participants were asked to recall the learning experience during the last week and then score the six statements on a Likert scale ranging from 1 (“strongly disagree”) to 7 (“strongly agree”). The higher the total score, the greater the engagement. The internal consistency estimated by Cronbach’s alpha coefficient for this sample was 0.93 at T1, 0.95 at T2, and 0.96 at T3, respectively.

##### Meaning in life

The Chinese version of the 5-item Meaning in Life Questionnaire Presence (MLQ-P)^3^ validated by Liu and Gan^49^ was employed to assess MIL in this study. This scale, which measures how meaningful one considers his/her life to be, has been found to be valid and internally reliable among Chinese samples (e.g., Refs.^50,51^). Participants were asked to rate the five statements (e.g., “I have a good sense of what makes my life meaningful” and “I have discovered a satisfying life purpose”) on a Likert scale ranging from 1 (“strongly disagree”) to 7 (“strongly agree”). Higher ratings represented a deeper extent to which individuals perceive the MIL. Similar to previous studies^52^, item 5 (“My life has no clear purpose”), yielded low factor loadings and low correlations with the scale and was removed from subsequent analyses^45^. The internal consistency estimated by Cronbach’s alpha coefficient for the final 4-item scale was 0.91, 0.93, and 0.93 at T1, T2, and T3, respectively.

#### Software

IBM SPSS 26.0 was used for description and correlation analysis, while Mplus 8.1^53^ was used to conduct confirmatory factor analysis (CFA) and test the CLPM.

### Procedure

First of all, ethical approval of the present work was obtained from the Ethics Committee of the first author’s institution. Besides, the measures assessing gratitude, LE, and MIL, were loaded on Jinshuju, a professional online platform for questionnaire distribution and data collection and distributed to a small sample for a pilot test to ensure further the translated versions maintained their accuracy and relevancy within the Chinese context.

Following prior literature^8–10^, in each week of the positive psychology course, participants wrote a gratitude note with the instruction, “Please list three people or things that you were grateful for last week”, in class. After the gratitude practice at the first (T1), fifth (T2), and ninth week of the course (T3), three waves of data were collected by completing the online questionnaire package consisting of three measures assessing gratitude, LE, and MIL. It took participants approximately 5–10 mins.

### Statistical analyses

Firstly, Common Method Variance (CMV), considered a primary source of measurement error that can compromise the validity of conclusions about measured relationships^54^, was tested using CFA. Although the impact of CMV can be mitigated by time lag^54^, for greater rigor, we still tested it in two ways: Harman’s single-factor CFA test and the unmeasured latent method construct (ULMC)^55,56^.

Secondly, we tested each study variable’s measurement invariance (MI). Testing MI, one of the prerequisites of longitudinal design^57^, can help determine whether the measures we use have the same meanings^58^ over time and, thus, whether the responses to items are comparable. The criteria (ΔCFI ≤ 0.010, supplemented by ΔRMSEA ≤ 0.015) recommended by Chen^58^ were adopted to determine whether an instrument is psychometrically equivalent across time.

Then, descriptive statistics and correlational analysis regarding three study variables were conducted.

Finally, the CLPM, constructed with three variables, gratitude, LE, and MIL, was analyzed using the bootstrapping method. Since the Chi-square test is sensitive to both sample size and data distribution^59^, a two-index presentation strategy recommended by Hu and Bentler^60^ was adopted for model assessment and comparison: SRMR < 0.08 supplemented by either CFI > 0.95 or RMSEA < 0.06.

### Ethics statement

This study adhered to the ethical principles of the Helsinki Declaration and received approval from the Ethics Committee at Southwest Jiaotong University (No. XL-20230730-0001). Informed consent was obtained from all participants.

## Results

### Common method variance

Two methodological strategies were applied to assess CMV in the study: Harman’s Single-factor CFA Test and ULMC. The results of CFAs are demonstrated in Table 1. As for Harman’s Single-factor CFA Test, a three-factor model (M1) was compared to a single-factor model (M2), with observed indicators from each scale loaded onto their respective latent variables. Fitting indices indicated that M1(T1: *χ*^*2*^ = 211.08, *df* = 74, CFI = 0.97, RMSEA = 0.06, SRMR = 0.03. T2: *χ*^*2*^ = 297.08, *df* = 74, CFI = 0.97, RMSEA = 0.08, SRMR = 0.03. T3: *χ*^*2*^ = 322.35, *df* = 74, CFI = 0.97, RMSEA = 0.08, SRMR = 0.03.) significantly fit the data better than M2 (T1: *χ*^*2*^ = 1463.77, *df* = 77, CFI = 0.70, RMSEA = 0.19, SRMR = 0.12. T2: *χ*^*2*^ = 1724.07, *df* = 77, CFI = 0.77, RMSEA = 0.21, SRMR = 0.08. T3: *χ*^*2*^ = 1858.89, *df* = 77, CFI = 0.78, RMSEA = 0.22, SRMR = 0.08.), suggesting no substantial CMV. Regarding an alternative strategy of ULMC, a bi-factor model (M3) was compared to M1, with the addition of a first-order method factor using all measures as indicators. Changes in CFI, RMSEA, and SRMR did not significantly improve M3 compared to M1(T1: ΔCFI = 0.02, ΔRMSEA = 0.01, ΔSRMR = 0.01. T2: ΔCFI = 0.01, ΔRMSEA = 0.01, ΔSRMR = 0.01. T3: ΔCFI = 0.00, ΔRMSEA = 0.00, ΔSRMR = 0.01.). Together, the results of both strategies indicated no serious CMV in the present study, suggesting that our study variables are distinct constructs.

**Table 1:** Results of CFAs for CMV Test.

Models		*χ*^*2*^* (df)*	Model fit indices			ModelComparison	ΔCFI	ΔRMSEA	ΔSRMR
			CFI	RMSEA	SRMR				
T1	Three-factor (M1)	211.08 (74)	0.97	0.06	0.03				
	Single-factor (M2)	1463.77 (77)	0.70	0.19	0.12	M2 vs. M1	0.27	0.13	0.09
	Bi-factor (M3)	133.58 (63)	0.99	0.05	0.04	M3 vs. M1	0.02	0.01	0.01
T2	Three-factor (M1)	297.08 (74)	0.97	0.08	0.03				
	Single-factor (M2)	1724.07 (77)	0.77	0.21	0.08	M2 vs. M1	0.20	0.13	0.05
	Bi-factor (M3)	210.92 (63)	0.98	0.07	0.04	M3 vs. M1	0.01	0.01	0.01
T3	Three-factor (M1)	322.35 (74)	0.97	0.08	0.03				
	Single-factor (M2)	1858.89 (77)	0.78	0.22	0.08	M2 vs. M1	0.19	0.14	0.05
	Bi-factor (M3)	277.83 (63)	0.97	0.08	0.02	M3 vs. M1	0.00	0.00	0.01

*χ*^2^, Chi-square; *df*, degree of freedom; CFI, Comparative Fit Index; RMSEA, Root mean square error of approximation; SRMR, Standardized root mean square residual.

### Longitudinal measurement invariance

To examine MI, three successive models—configural invariance, metric invariance, and scalar invariance—were estimated for each study variable. Table 2 presents the fit indices for these models. Overall, the models exhibited a satisfying fit for the data. Importantly, the changes in CFI and RMSEA (gratitude: ΔCFI = 0.008, ΔRMSEA = 0.005. LE: ΔCFI = 0.002, ΔRMSEA = 0.001. MIL: ΔCFI = 0.000, ΔRMSEA = 0.004) suggested that all constructs were equivalent across time at both the configural and metric levels, while MIL further satisfied the criteria for scalar invariance (ΔCFI = 0.000, ΔRMSEA = 0.003).

**Table 2:** Model fit indices for MI test.

Models	*χ*^*2*^	*df*	Model fit indices		ΔCFI	ΔRMSEA
			CFI	RMSEA		
Gratitude						
Configural invariance	170.37	39	0.976	0.084		
Metric invariance	225.28	47	0.968	0.089	0.008	0.005
Scalar invariance	307.83	53	0.954	0.100	0.014	0.011
LE						
Configural invariance	366.28	114	0.972	0.068		
Metric invariance	394.71	126	0.970	0.067	0.002	0.001
Scalar invariance	525.83	135	0.956	0.078	0.014	0.011
MIL						
Configural invariance	77.67	39	0.992	0.045		
Metric invariance	84.55	47	0.992	0.041	0.000	0.004
Scalar invariance	90.42	53	0.992	0.038	0.000	0.003

*χ*^2^, Chi-square; *df*, degree of freedom; CFI, Comparative fit index; RMSEA, Root mean square error of approximation.

### Descriptive statistics and bivariate correlations

Table 3 presents the means, standard deviations, and bivariate correlations for study variables across time. As indicated, gratitude, LE, and MIL at T1 were significantly related to their counterparts at T2 (e.g., gratitude at T1 with gratitude T2,* r* = 0.44, *p* < 0.01) and T3 (e.g., MIL at T1 with MIL at T3,* r* = 0.46, *p* < 0.01). Furthermore, all variables showed significant positive correlations with each other across time (e.g., gratitude at T1 with LE at T1, *r* = 0.37, *p* < 0.01).

**Table 3:** Descriptive statistics and bivariate correlations.

Variables	Bivariate correlations (*N* = 479)								
	1	2	3	4	5	6	7	8	9
1 Gratitude (T1)	1								
2 LE (T1)	0.37**	1							
3 MIL (T1)	0.39**	0.65**	1						
4 Gratitude (T2)	0.44**	0.41**	0.37**	1					
5 LE (T2)	0.39**	0.49**	0.42**	0.69**	1				
6 MIL (T2)	0.36**	0.47**	0.52**	0.68**	0.78**	1			
7 Gratitude (T3)	0.45**	0.39**	0.33**	0.80**	0.63**	0.57**	1		
8 LE (T3)	0.35**	0.44**	0.35**	0.57**	0.70**	0.61**	0.67**	1	
9 MIL (T3)	0.32**	0.45**	0.46**	0.62**	0.67**	0.70**	0.70**	0.82**	1
*M*	22.82	28.21	19.23	21.54	29.37	19.56	21.29	29.19	19.80
*SD*	4.43	7.69	5.19	5.06	7.78	5.17	5.44	8.50	5.28

LE, Learning engagement; MIL, Meaning in life. **p < 0.01.

### Test for cross-lagged panel model

**Figure 2:**
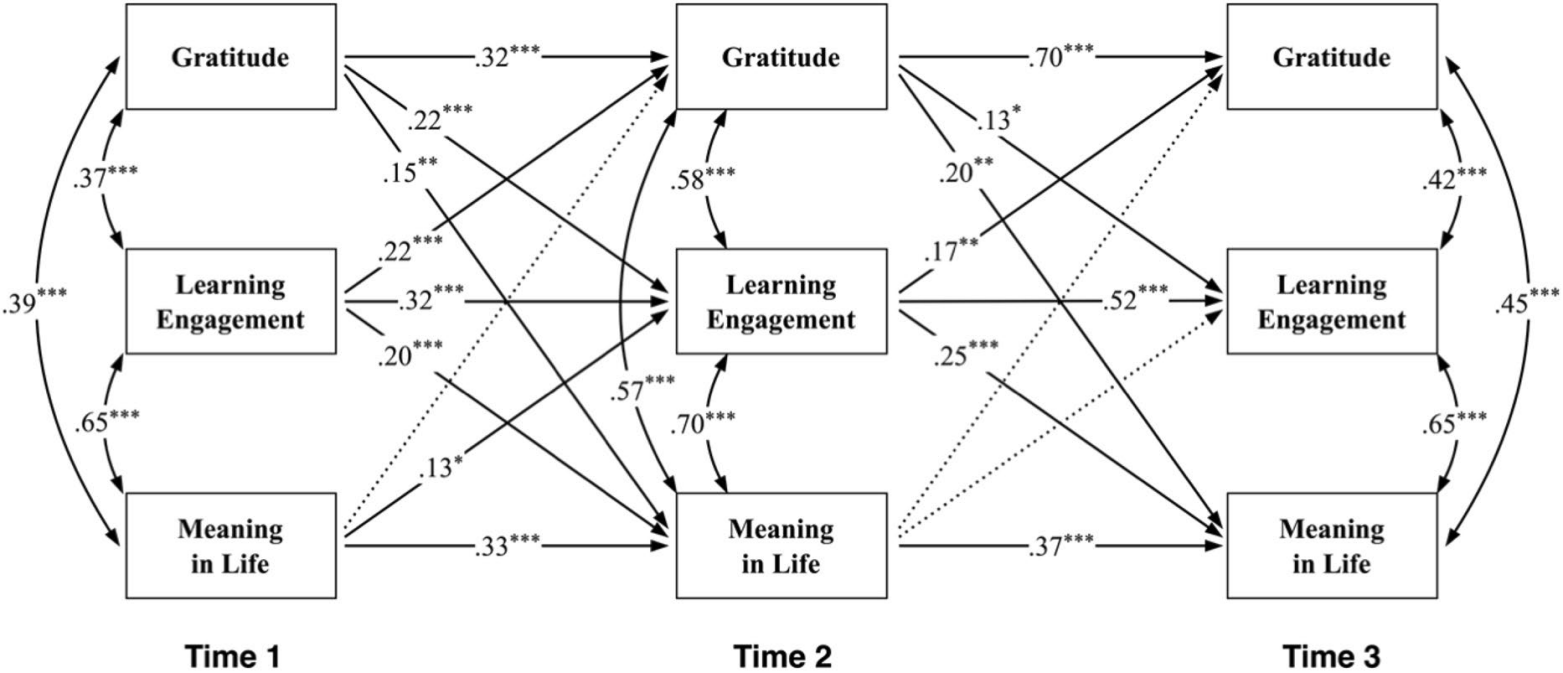
Final model (M4) with standardized path coefficients. *Note*. Solid lines represent the significant paths, while dotted lines represent the non-significant paths. *p < 0.05, ** p < 0.01, *** p < 0.001.

To examine the causal ordering between gratitude and MIL, as well as the mediating effect of LE, four competing models were constructed and tested against one another: M1 = all auto-regressive paths and the covariance correlations of variables at the same time point; M2 = M1 + cross-lagged paths from gratitude to MIL, gratitude to LE, and LE to MIL within one-time unit; M3 = M1 + cross-lagged paths from MIL to gratitude, MIL to LE, and LE to gratitude within one-time unit; M4 = M1 + M2 + M3. It is worth noting that the paths from MIL to gratitude were included in M3 and M4 to check for reversed causality. The fit indices suggested that M4 (*χ*^2^ = 44.57, *df* = 9, CFI = 0.99, RMSEA = 0.09, SRMR = 0.02) fitted the data better than the three alternative models, thereby being adopted as the final model (see Fig. 2).

As shown in the final model, all autoregressive paths were significant. Within the one-time unit, the lagged effect of gratitude at T1 on LE (*β* = 0.22, *p* < 0.001) and MIL at T2 (*β* = 0.15, *p* < 0.01), and gratitude at T2 on LE (*β* = 0.13, *p* < 0.05) and MIL at T3 (*β* = 0.20, *p* < 0.01) were positive and significant. Whereas among the reversed effects (from MIL), the only significant lagged effect was found from MIL at T1 to LE at T2 (*β* = 0.13, *p* < 0.05). The results suggested that gratitude had a positive lagged effect on MIL, whereas MIL didn’t have much effect on gratitude. In addition, LE at T1 positively associated with both gratitude (*β* = 0.22, *p* < 0.001) and MIL at T2 (*β* = 0.20, *p* < 0.001), and LE at T2 positively associated with gratitude (*β* = 0.17, *p* < 0.01) and MIL at T3 (*β* = 0.25, *p* < 0.001).

Table 4 displays the indirect paths in the final model. The path from gratitude at T1 to MIL at T3 via LE at T2 was significant (*β* = 0.05, *SE* = 0.02, 95% CI = [0.02, 0.10]). However, the reverse path (from MIL at T1 to gratitude at T3 via LE at T2) was insignificant due to the inclusion of 0 in the 95% CI (*β* = 0.02, *SE* = 0.01, 95% CI = [0.00, 0.05]). The results indicated that gratitude at T1 positively predicted MIL at T3 via LE at T2. However, there was no sufficient evidence suggesting that LE at T2 played a mediating role in the relationship between MIL at T1 and gratitude at T3.

**Table 4:** Standardized indirect effects with 95% confidence intervals (CI).

Paths	*β*	*SE*	95% CI	
			Lower 2.5%	Upper 2.5%
T1 Gratitude → T2 LE → T3 MIL	0.05	0.02	0.02	0.10
T1 MIL → T2 LE → T3 Gratitude	0.02	0.01	0.00	0.05

LE, learning engagement; MIL, meaning in life.

## Discussion

The study, building on theories of the “find-remind-and-bind,” SDT, and broaden-and-build model, conducted analyses across the three-time waves and aimed to provide a more nuanced understanding of how gratitude, LE, and MIL interact over time among university students following earthquakes, contributing to the existing literature by deepening our insights into the intricate interactions among these psychological constructs, specifically within the context of university students navigating the aftermath of severe earthquakes.

### General discussion on the autoregressive effect

Firstly, our autoregression analysis underscores the inherent stability of gratitude, LE, and MIL among university students. This continuity indicates that past levels of gratitude, LE, and MIL are reliable predictors of their future states. For instance, students who have previously displayed high levels of gratitude and LE will likely remain in the state over time, culminating in enhanced MIL^61^. This finding emphasizes the importance of consistently fostering the practice of gratitude to harvest cumulative benefits over time. Given the ongoing challenges students face, particularly those affected by the trauma of earthquakes, cultivating these positive attributes becomes even more crucial in promoting their resilience and well-being amidst adversity^1^. The results highlight the potential of gratitude and LE as protective factors, which, when nurtured over time, may contribute to a greater sense of MIL, offering valuable implications for supporting students’ psychological growth and adjustment in the aftermath of the earthquake.

### The cross-lagged effect between gratitude and meaning in life

Our three-wave longitudinal design examines the predictive positive relationship between gratitude and MIL, contributing to a better understanding of how these two factors interact over time. Gratitude, recognized as a positive emotion, directs individuals’ attention toward the positive aspects of their existence and surroundings^18^. This shift in focus towards the beneficial aspects can promote personal and communal well-being, leading to a heightened sense of purpose and MIL^22^.

Our findings echo the prior study suggesting that gratitude practice potentially strengthens the inclination to cultivate a meaningful existence and is positively associated with a sense of life’s purpose^19^. Viewed as a life orientation, gratitude is cognitively correlated with MIL^21^. Additionally, gratitude and MIL have consistently been identified as significant contributors to positive outcomes such as innovative behavior and overall well-being^3^. Our research aligns with prior findings that gratitude can enhance students’ perception of MIL after the earthquake^7^, but also extends beyond by longitudinally exploring the impact of gratitude on MIL. The results support Algoe’s “find-remind-and-bind” theory^25^, suggesting that gratitude enhances our sense of belonging and interconnectedness—fundamental components of MIL^62^.

The implications of this finding are particularly significant for university students who have experienced traumatic events such as earthquakes, which can deeply disrupt their sense of meaning and purpose in life^1^. However, our study highlights gratitude as a potential protective factor that can enhance students’ perception of MIL even in adversity^7^. By cultivating gratitude, students may appreciate positive life events, nurture their relationships with teachers and peers, and foster a sense of belonging. Consequently, they can respond more resiliently and meaningfully to challenges.

The implications of this finding also emphasize the importance of promoting gratitude as a crucial element in enhancing students’ sense of meaning, supporting previous research^24^. These results underscore the need for targeted interventions and support networks that cultivate a gratitude mindset among university students, especially in the aftermath of traumatic events like earthquakes. Educators and mental health professionals can provide essential resources and guidance to help students navigate obstacles and promote their psychological well-being through gratitude practices, such as recognizing each other’s strengths, journaling gratitude, and expressing appreciation to others.

### The path effect of gratitude on meaning in life via learning engagement

Our findings provide compelling evidence that gratitude significantly and positively affects MIL over time through learning engagement among university students affected by earthquakes. This is in accordance with previous findings which reported that traumatic events can cause significant disruption to students’ studies for weeks to months or longer^63^, and prompt them to reevaluate their lives^64^. It also confirmed what Jin and Wang^65^ discovered, that gratitude significantly predicted adolescents’ learning engagement through the fulfillment of basic psychological needs. Therefore, this finding aligns with existing research on SDT, which emphasizes the significance of fulfilling basic psychological needs (through pathways like gratitude), including autonomy and competence, to foster intrinsic motivation and engagement in learning activities^66^, which in turn contributes to a heightened sense of MIL in the context of recovery from traumatic events such as earthquakes. In addition, our methods adopted to explore such a relationship are advocated by Kim and Oh^67^ who believed that longitudinal studies can be able to capture the evolution of posttraumatic growth more accurately over time.

Moreover, gratitude, recognized as a psychological catalyst of LE^65^, can amplify LE by broadening students’ cognitive horizons, aligning with the broaden-and-build theory of positive emotions^35,36, 68^. This cognitive expansion may foster deeper engagement in academic pursuits, while the resources built through this process can provide resilience in the face of academic adversities. Engaged learners are more likely to experience positive outcomes, such as enhanced academic performance and satisfaction^65^, which, in turn, gratify their basic psychological needs, particularly the need for competence, thereby elevating MIL^51,69^. To foster a deeper engagement in learning, educators can encourage students to set personal goals related to their interests, facilitating motivation and self-efficacy. A supportive environment that promotes autonomy and collaboration can create a safe space for intellectual expression and a sense of community, essential for sustained engagement and a meaningful educational experience. These positive outcomes may also contribute to cultivating self-understanding and affirming one’s self-value, enriching the feeling of life’s meaningfulness^26^.

In summary, the mediating effect of LE is detected in the relationship between gratitude and MIL in our longitudinal cross-lagged design. This pioneering research provides valuable theoretical insights as it is the first study to empirically demonstrate the temporal dynamics among the three variables. In practical terms, educators and mental health professionals can leverage these findings to design interventions that encourage students to cultivate a greater recognition of the importance of small acts of kindness during difficult times, to enhance their LE, fostering meaning in life.

### Limitations and future directions

Despite the notable findings, our study is not without limitations. Firstly, as is common in many studies employing self-report measures, our research might be subject to certain inherent biases such as recall bias (where participants may inaccurately remember their past experiences) and social desirability bias (where participants might respond in a manner they perceive to be socially acceptable rather than their true feelings or behaviors). To overcome such limitations, future research might benefit from employing behavioral observation or peer reports, which could supplement self-report data and provide a more comprehensive view of the constructs under study.

Secondly, it is essential to note that the temporal structure of our three-wave longitudinal study, may not adequately capture the long-term dynamic effect among the interplay of the constructs. Future research, therefore, should consider adopting more extended or variable time intervals (e.g., three-month, six-month, or one-year intervals) to gain a more nuanced understanding of the temporal causal effects enriching our understanding of the intricate interplay between gratitude, LE, and MIL.

Thirdly, the absence of a control group consisting of individuals who did not experience the earthquake and the lack of data on individual distress levels presents challenges in drawing definitive conclusions regarding whether the observed outcomes primarily reflect the effects of gratitude or its stress-buffering capacities. We encourage future research to expand upon our findings and delve deeper into these potential avenues for investigation.

## Data Availability

The datasets generated for this study are fully available upon reasonable request to the first or corresponding author.
